# Oxidative stress parameters of Gaucher disease type I patients

**DOI:** 10.1016/j.ymgmr.2015.05.001

**Published:** 2015-05-22

**Authors:** Alexandre Silva Mello, Cristina da Silva Garcia, Fernanda de Souza Machado, Niara da Silva Medeiros, Mariane Farias Wohlenberg, Jéssica Pereira Marinho, Caroline Dani, Cláudia Funchal, Janice Carneiro Coelho

**Affiliations:** a Research Center, Methodist University Centre — IPA, Porto Alegre, Brazil; b Department of Biochemistry, ICBS-UFRGS, Federal University of Rio Grande do Sul, Porto Alegre, Brazil

**Keywords:** β-Glucosidase, Reactive oxygen Species, Gaucher disease type I, Lysosomal storage disorders, Oxidative stress

## Abstract

Enzymatic deficiency in Gaucher disease (GD) patients may induce a cascade of events, culminating in secondary effects such as the production of reactive oxygen species (ROS). Detoxification through biological systems which remove or repair the damage may cause the production of peroxides and free radicals that damage all cell components, including proteins, lipids and ADN. The study's aim was the test, using the analysis of plasma samples' the use of lipid peroxidation by thiobarbituric acid reactive substances (TBARS), protein damage by carbonyl assay, non-enzymatic antioxidant defenses by sulfhydryl (SH) content, antioxidant enzymatic defenses by catalase (CAT) and superoxide dismutase (SOD), from patients with GD type I patients who received no prior treatment. Blood samples were collected from 10 patients previously diagnosed with GD type I and from 11 healthy subjects. Chitotriosidase (CT) activity was measured in plasma and the activity of β-glucosidase (GBA) was measured in leukocytes. The results showed a significant increased (*p* < 0.005) in GD samples when compared to healthy controls in CAT, SOD and SH, but there was no change in TBARS and carbonyl in the comparison between the two groups. In conclusion, the present data indicates the increased levels of enzymatic and non-enzymatic defenses without any effect on lipid peroxidation and damage to proteins. We believe that the results of this study are relevant to understanding the cellular changes involved in this important LSDs.

## 1 Introduction

Lysosomal storage disorders (LSDs) represent a group of more than 50 different inherited metabolic diseases resulting from defective function of the specific lysosomal enzyme, or defects in non-enzymatic lysosomal or non-lysosomal proteins. Due to the progressive accumulation of metabolites not degraded in lysosomes, a cellular and widespread tissue dysfunction (in addition to multisystem disorders) occurs [1]. Most LSDs are autosomal recessive origin; these diseases are rare, with a combined incidence estimated at 1 in 5000 live births [2], [3].

GD is a LSD where a storage of glucosylceramide (GlcCer) occurs due to deficiency of the lysosomal enzyme glucocerebrosidase, causing multiple organ dysfunctions [4], [5]. The enzyme is present in the lysosomes of all nucleated cells and cleaves the β-glucosidic bond of GlcCer, yielding glucose and ceramide [6], [7]. The disease can be classified in three clinical types. Type I, the most common, is the chronic, non-neuropathic form of the disease, which shows highly variable signs and symptoms and a variable course, with visceral and skeletal involvement (splenomegaly, hepatomegaly and bone damage that might lead to fractures) and hematologic anomalies (pancytopenia), among others. The neurological involvement can be observed in types 2 and 3 [8].

Evidence shows that the storage of GlcCer in macrophages is associated with inflammatory processes and the production of a reactive species [9], [10]. Enzymatic deficiency in GD patients may induce a cascade of events that results in side effects, such as the production of reactive oxygen species (ROS) and reactive nitrogen species (RNS) that can then generate the oxidative stress [9], [11], [12], whereas in the body of healthy individuals, the production and degradation of ROS and RNS are generally balanced [13].

Reactive species are naturally formed during biological metabolism, but our organism is also capable of developing an antioxidant defense system, which may be enzymatic or non-enzymatic [14].

Oxidative stress occurs when there is an imbalance between pro-oxidants and antioxidants, in favor of pro-oxidants [15]. Some studies describe the relationship between Inborn Errors of Metabolism (IEM) and oxidative stress, but most of these studies assess the efficiency of enzyme replacement therapy, and not intracellular changes caused by ROS in affected patients of GD [16], [17].

**Fig. 1:**
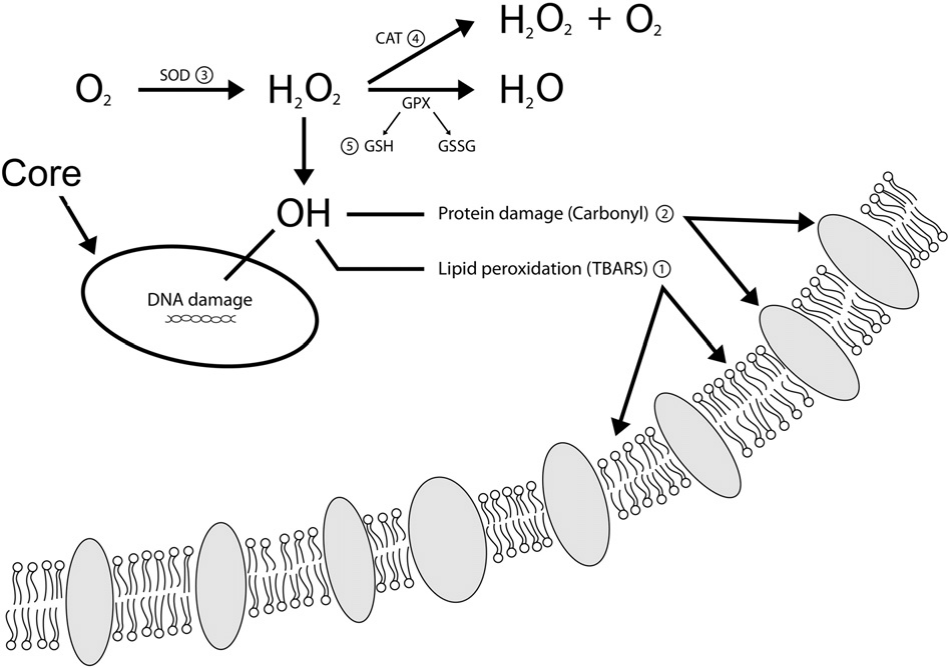
Overview of oxidative damage markers and antioxidant defense markers used in this study: 1. thiobarbituric acid reactive substances (TBARS); 2. carbonyl; 3. superoxide dismutase (SOD); 4. catalase (CAT); 5. total sulfhydryl (SH) content.

This study aimed to test the use of thiobarbituric acid substances (TBARS) and carbonyl as markers of oxidative damage, in addition to the catalase (CAT), superoxide dismutase (SOD) and total content of sulfhydryl (SH) as markers of antioxidant defense measured in plasma to better understand the cellular changes in GD patients (Fig. 1). Therefore, we investigated the relation between ROS and GD, analyzing markers of oxidative stress in the blood of patients with GD type I, compared with the blood from healthy controls (HC).

## 2 Methods

### 2.1 Patients and controls

Blood samples of 9 mL were collected directly from 10 patients previously diagnosed with GD type I, and from 11 healthy subjects by one of the authors of this work. This study included 10 patients (7 women and 3 men; 3–46 years old) with DG and 11 HC (4 women and 7 men; 3–60 years old). For donors older than 18 years old, or those responsible for underage donors, an informed consent was obtained according to the guidelines of the committee.

All samples were identified with numbers, preserving the identity of donors, who were not informed of the results nor had their identities revealed at any stage of the procedure. As a criterion for inclusion of the samples individuals had to be at least 7 years old, weigh more than 18 kg and be higher than 90 cm.

The sample size calculation for comparing averages with different variances was made, establishing the level of significance at 5% and power by 90%. For this we used the MiniTab® 15 statistical software. The calculation indicated 10 subjects per group for a total of 20 samples.

The total heparinized blood was centrifuged to separate plasma and leukocytes and underwent a separation technique [18]. The chitotriosidase (CT) activity was measured in plasma, according to the technique of Hollak et al. [19] and β-glucosidase (GBA) was activity measured in leukocytes, according to the technique of Goldim et al. [20]. Reference values were established by assessing blood samples provided by the healthy volunteer donors. The diagnosis of GD patients was made in the Federal University of Rio Grande do Sul (Porto Alegre, RS, Brazil).

Lipid peroxidation and protein damage was analyzed using samples of plasma, the former through TBARS and the latter through carbonyl assay. The content of the non-enzymatic antioxidant defenses (SH) and the activity of the antioxidant enzymatic defenses CAT and SOD were also analyzed.

The research protocols and consent forms, as well as the investigation were ethically and scientifically approved by the Research and Ethical Committee of UFRGS (no. 25686).

### 2.2 Enzymatic activities

#### 2.2.1 β-Glucosidase (GBA) activity

Reference values were established by assessing blood samples provided by healthy volunteer donors. GBA leukocyte activities [21], [22] were measured using artificial 4-methylumbellipheryl substrate (Sigma, St. Louis, MO, USA) in dilution buffer (0.54 M citrate phosphate, pH 5.5 for GBA). Reactions were stopped using 0.5 M glycine–NaOH, pH 10.3 (GBA). After incubation, 200 μL aliquots were transferred to black 96 well plates (PerkinElmer, 96F). All incubations were done at 37 °C in dry plates with shaking (Marconi MA-127). GBA activity measurements were in standard technique [23]. Standard analysis was carried out in 1.5 mL plastic tubes (Eppendorf). All readings were performed at 365 nm (excitation) and 450 nm (emission) in a 96 well plate reader (Spectra Max M5, Molecular Devices).

#### 2.2.2 Chitotriosidase (CT) activity

A technique used for measuring enzyme activity in plasma CT was described by Hollak et al. [19]. Reactions were stopped using 0.13 methylenediamine, pH 11.3 (CT). New reference values and cutoff values were established using ROC curve with 100% sensitivity and specificity. All readings were performed at 365 nm (excitation) and 450 nm (emission) in a 96 well plate reader (Spectra Max M5, Molecular Devices).

### 2.3 Parameters of oxidative stress

#### 2.3.1 Thiobarbituric acid reactive substances (TBARS)

As an index of lipid peroxidation, we used TBARS production during an acid-heating reaction, which is widely adopted as a sensitive method for measuring lipid peroxidation, as previously described by Wills [24]. The samples were stirred for a brief period of time with 10% trichloroacetic acid (TCA) and 0.67% thiobarbituric acid (TBA) and then heated in a boiling water bath for 15 min in closed tubes. TBARS were determined by absorbance at 535 nm. Results were expressed as nmol/mg of protein.

#### 2.3.2 Measuring levels of oxidatively modified proteins

Oxidative damage to proteins was assessed by determining carbonyl groups on the basis of its reaction with dinitrophenylhydrazine (DNPH), as previously described by Levine et al. [25]. DNPH reacts with protein carbonyls to form hydrazones, which can be measured spectrophotometrically. Firstly, 500 μL 10 mM DNPH in 2 M HCl was added at room temperature, for 1 h, with vortexing every 10–15 min. Next, 500 μL 20% TCA was added, and tubes were mixed and centrifuged for 3 min. The supernatant was then discarded, and the pellets were washed three times with 1 mL of ethanol–ethyl acetate (1:1) to remove free reagent. After centrifugation, the precipitated protein was re-dissolved in 0.6 mL guanidine solution. Proteins were dissolved within 15 min at 37 °C. The insoluble material was removed by centrifugation in a microcentrifuge for 3 min. The absorbance was read at 370 nm. Equal amounts of protein samples without DNPH were used as controls. The results were expressed in nmol/mg of protein.

#### 2.3.3 Measurement of total sulfhydryl (SH) groups

Sulfhydryl assay is based on the reduction of 5,5′-dithio-bis(2-nitrobenzoic acid) (DTNB) by thiols, generating a yellow derivative (TNB) whose absorption is measured spectrophotometrically at 412 nm. 0.1 mM DTNB was added to 120 μL of the samples. This was followed by a 30-minute incubation at room temperature in a dark room. Absorption was measured at 412 nm. The sulfhydryl content is inversely correlated to oxidative damage to proteins. Results were reported in nmol/mg protein [26].

#### 2.3.4 Measure of antioxidant enzyme activity

SOD activity was determined by a spectrophotometric method, measuring the inhibition of the rate of adrenochrome formation at 480 nm (Spectrophotometer SP-2200, Bioespectro) in medium containing 1 mM adrenalin and 50 mM glycine [27]. The results were expressed in USOD/mg protein. The method used to determine CAT activity has been described by Aebi [28] and determines the rate of H_2_O_2_ degradation, measuring absorbance at 240 nm (Spectrophotometer SP-2200, Bioespectro). The results were expressed as UCAT/mg protein.

### 2.4 Protein determination

Protein concentration was determined according to the method described by Lowry et al. [29].

### 2.5 Statistical analysis

The data were evaluated through the Student's t test, followed by Levene's test, used to compare the results of the analysis of plasma with those of both HC and GD patients. Analysis was performed using the statistical software package SPSS, version 17.0 (SPSS Inc., Chicago, IL, USA), and level of significance was set at *p* < 0.05. Data are reported as mean ± SE.

## 3 Results

A total of 10 GD patients and 11 HC were included in this report. The average age was 20.00 years ± 15.69 for DG and 25.18 years ± 18.91 for HC. After sample collection we measured enzymatic analysis, to confirm that a sample was from an HC or a GD patient. Values were significantly different between the two enzymes in the groups included in this study, with GBA showing increased values in the HC group (15.74 ± 6.60 [HC] and 1.20 ± 0.80 [GD]; *p* = 0.001), whereas CT enzyme levels were higher in the GD group (16,720.96 ± 18,462.67 [GD] and 50.23 ± 44.17 [HC]; *p* = 0.019).

**Fig. 2:**
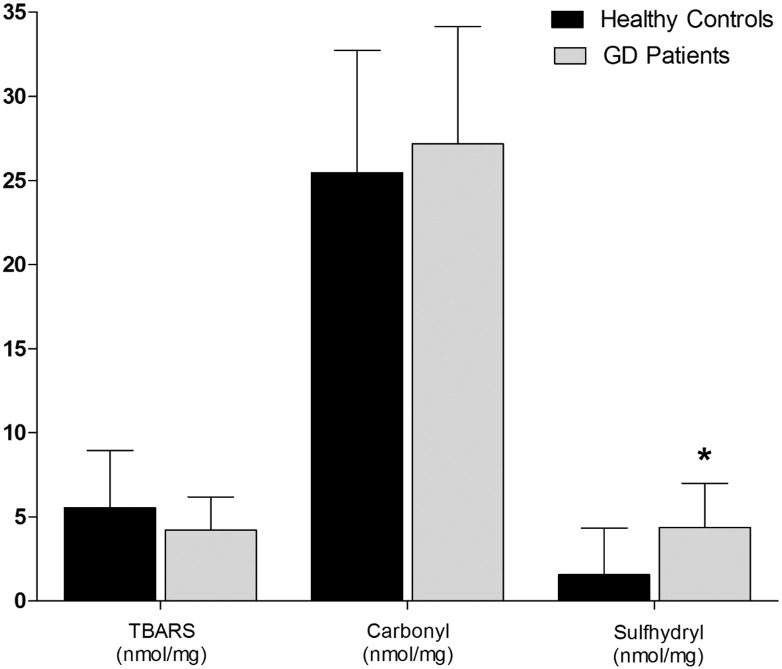
Oxidative damage to proteins and lipids: TBARS and carbonyl; and sulfhydryl in the plasma of GD and HC. Data are expressed as mean ± SD. *Different letters indicate statistical differences according to ANOVA and Tukey's post-test (*p* < 0.05).

We did not observe statistical differences between HC and GD groups for TBARS and carbonyl assays (Fig. 2). However, the results showed a statistical difference between the groups for SH (*p* < 0.04) with 1.57 ± 2.77 nmol/mg for HC and 4.38 ± 2.62 nmol/mg for GD patients (Fig. 2), showing higher levels for GD.

**Fig. 3:**
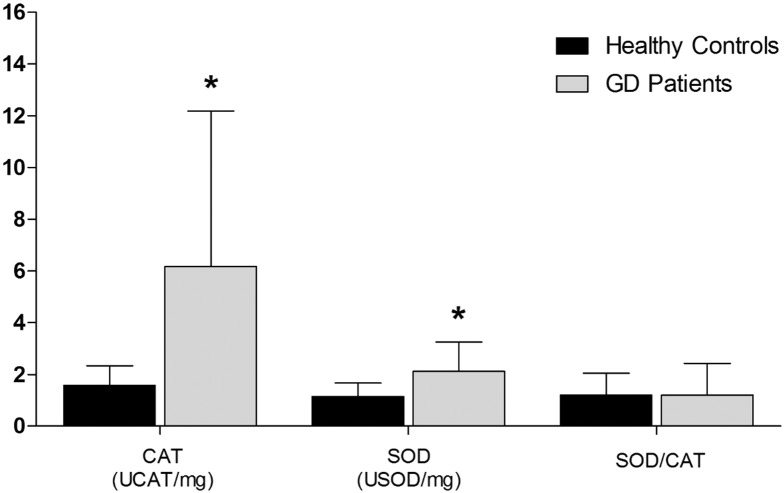
Enzymatic and non-enzymatic activities: CAT; SOD and SOD/CAT radio in the plasma of GD and HC. Data are expressed as mean ± SD. *Different letters indicate statistical differences according to ANOVA and Tukey's post-test (*p* < 0.05).

The mean between groups were significantly different in the CAT activity (*p* < 0.03); 1.58 ± 0.76 UCAT/mg for HC and 6.18 ± 6.01 UCAT/mg for GD patients (Fig. 3). The mean SOD activity (*p* < 0.04) was 1.15 ± 0.52 USOD/mg for HC and 2.12 ± 1.13 USOD/mg for GD patients (Fig. 3). In addition, we observed statistical differences in SOD and CAT activities (*p* < 0.03 and *p* < 0.04, respectively) in the GD group, which showed higher levels than the control group. However, we did not observe differences in SOD/CAT ratio (Fig. 3).

## 4 Discussion

IEM results from the lack of or deficiency in the activity of specific enzymes or proteins, leading to an accumulation of metabolic intermediates. The incidence of IEM is rare in the population in general, 1:1000 live births [30].

GD is considered a lysosomal storage disease in which the deficiency of GBA enzyme leads to the accumulation of GlcCer in lysosomes of the reticuloendothelial system. The cause of GBA deficiency may be associated with a mutation in an allele of the gene encoding the enzyme, but little is known about the mechanisms that lead to tissue damage [31], [32], [33]. Evidence shows that the storage of GlcCer in macrophages is associated with inflammatory processes and the production of reactive species [9].

In a recent study, Paspalj et al. were able to perform an analysis of the relation between redox disturbance and clinical outcome in patients with acute ischemic stroke, which may be of interest to elucidate the molecular mechanisms involved in this life-threatening condition [34].

Several studies suggest that the presence of redox impairment may play a role in the pathogenesis of GD [11], [12], [13]. Many studies have correlated IEM with increased ROS and antioxidant deficiency, which contribute to oxidative stress. Evidence suggests that oxidative stress may be an important pathological factor for numerous IEM [35], [36], [37], [38], [39]. Literature shows the deficiency of the enzyme GBA in cultured human fibroblasts increases the amount of reactive species [12]. Another study shows that GBA deficiency in patients alters the activity of the antioxidant enzymes catalase and superoxide dismutase in erythrocytes [11].

Results showed what seems to be a chronic condition control which is characteristic in the group of GD type I patients when compared to healthy controls, also showing the highest values of SH in GD compared to healthy controls, indicating that a larger amount of the SH group activity seems to be directly related to the control of lipid peroxidation.

Glutathione (GSH) has a central role in protecting cells against oxidative stress [11], [40]. Many of the reactions of GSH involving the sulfhydryl group are highly polarizable. These thiols are a class of organic sulfur derivatives, characterized by the presence of sulfhydryl residues present in biological systems, in addition to various functions including a central role in coordinating the antioxidant defense network. The mammalian biological system is a system that regulates the cellular redox state of SH, protecting cellular protein, which contains excessive oxidation of SH. This system includes low molecular weight in the SH donor groups and enzymes which can catalyze the reduction of the SH groups of proteins and pro-oxidants [41].

One group used plasmalogens, which represent a unique class of phospholipids. Reduced red blood cell plasmalogen levels were reported in GD patients. The relation between plasmalogen abnormalities in GD patients and malonyldialdehyde levels (an indicator of lipid peroxidation) and the total antioxidant status, was further investigated [42]. No significant difference in the levels of plasma TBARS (a marker of lipid peroxidation) was found, which is a strong indicator of oxidative stress within the cell. The data found in TBARS, corroborated by the literature, shows no difference in the values between the groups studied [11].

Detection of carbonyl groups is being widely used as an indicator of oxidative damage to proteins [43]. The increase in the protein carbonyl group is associated with numerous pathological disorders, including rheumatoid arthritis, Alzheimer's disease, respiratory distress syndrome, Parkinson's disease and atherosclerosis [44]. However, we have seen no change between the values of this parameter, showing another form of control that the cells of GD patients seem to exhibit as opposed to the condition of chronic infection of the diseases mentioned before.

The SOD and CAT enzymes are among the cellular enzymatic antioxidants studied, which provide the first endogenous line of defense against oxidative injury. These alterations in the antioxidant system may cause the accumulation of H_2_O_2_ or products of its decomposition present not only in the cytosol, but also in mitochondria, the production site of much of the reactive species B [14], [15], [45].

Firstly, our results corroborate other studies that have shown an increase in the amount of activity in the CAT antioxidant enzyme in patients when compared to healthy controls [11], not only in GD, as well as in other lysosomal storage diseases (DLDs): Mucopolysaccharidosis type I and Fabry's disease [11], [46], [47]. However, we have found a significant difference between groups in SOD, with the opposite results to other research groups, who obtained lower values for GD when compared to the healthy control group [11], [48], [49]. This can be explained by controlling the levels of superoxide anion, as the increase in the levels of SOD in GD patients compared to healthy controls would avoid a change in oxidation processes, such as protein damage. However, through the analysis of carbonyl, a marker of protein damage, no significant change was observed [13].

Furthermore, even though the monocyte/macrophage GD is chronically activated [50], they show a reduction in the generation of oxidative stress [51]. This may be due to monocyte GD, which adapts to survive under basal conditions, oxidative stress, in a state of chronic activation. Under these conditions, the cells become unable to be activated in response to an antigenic stimulus [12].

Our work shows what appears to be an intracellular control, once our results show an increase in CAT, SOD and SH (intracellular standard markers), whereas TBARS and carbonyl (cell membrane markers) showed no difference between groups. In conclusion, the present data indicates the increased value of enzymatic and non-enzymatic defenses, without any effect on lipid peroxidation and damage to proteins.

## 5 Conclusion

Our study showed an alteration in CAT, SOD and SH, which suggests that there was a change in reactive oxygen species in GD type I patients when compared to HC. This increase in CAT, SOD and sulfhydryl could likely be related to the prevention of the increase of hydrogen peroxide, preventing damage to lipids, confirmed by the TBARS and carbonyl value maintenance. However, the other three parameters (TBARS, carbonyl and SOD/CAT ratio) did not show a significant difference after conducting statistical analysis. We believe that the results of this study are significant to understanding the cellular changes involved in this important LSDs.

## Conflict of interest

The authors declare no conflicts of interest.
